# Contribution of severe mental disorders to fatally harmful effects of physical disorders: national cohort study

**DOI:** 10.1192/bjp.2024.110

**Published:** 2024-10

**Authors:** Tomáš Formánek, Dzmitry Krupchanka, Benjamin I. Perry, Karolína Mladá, Emanuele F. Osimo, Jiří Masopust, Peter B. Jones, Oleguer Plana-Ripoll

**Affiliations:** Department of Psychiatry, University of Cambridge, Cambridge, UK; and Department of Public Mental Health, National Institute of Mental Health, Klecany, Czech Republic; Department of Mental Health and Substance Use, World Health Organization, Geneva, Switzerland; Department of Psychiatry, University of Cambridge, Cambridge, UK; and Cambridgeshire and Peterborough NHS Foundation Trust, Cambridge, UK; Department of Public Mental Health, National Institute of Mental Health, Klecany, Czech Republic; and Department of Psychiatry, Faculty of Medicine in Pilsen, Charles University, Pilsen, Czech Republic; Department of Psychiatry, University of Cambridge, Cambridge, UK; Cambridgeshire and Peterborough NHS Foundation Trust, Cambridge, UK; Institute of Clinical Sciences, Imperial College, London, UK; MRC London Institute of Medical Sciences, London, UK; and South London and Maudsley NHS Foundation Trust, London, UK; Department of Psychiatry, University Hospital Hradec Králové, Hradec Králové, Czech Republic; and Faculty of Medicine, Charles University, Hradec Králové, Czech Republic; Department of Clinical Epidemiology, Aarhus University, Aarhus, Denmark; Aarhus University Hospital, Aarhus, Denmark; and National Centre for Register-based Research, Aarhus University, Aarhus, Denmark

**Keywords:** Psychotic disorders/schizophrenia, bipolar type I or II disorders, depressive disorders, mortality and morbidity, comorbidity

## Abstract

**Background:**

It remains unknown whether severe mental disorders contribute to fatally harmful effects of physical illness.

**Aims:**

To investigate the risk of all-cause death and loss of life-years following the onset of a wide range of physical health conditions in people with severe mental disorders compared with matched counterparts who had only these physical health conditions, and to assess whether these associations can be fully explained by this patient group having more clinically recorded physical illness.

**Method:**

Using Czech national in-patient register data, we identified individuals with 28 physical health conditions recorded between 1999 and 2017, separately for each condition. In these people, we identified individuals who had severe mental disorders recorded before the physical health condition and exactly matched them with up to five counterparts who had no recorded prior severe mental disorders. We estimated the risk of all-cause death and lost life-years following each of the physical health conditions in people with pre-existing severe mental disorders compared with matched counterparts without severe mental disorders.

**Results:**

People with severe mental disorders had an elevated risk of all-cause death following the onset of 7 out of 9 broadly defined and 14 out of 19 specific physical health conditions. People with severe mental disorders lost additional life-years following the onset of 8 out 9 broadly defined and 13 out of 19 specific physical health conditions. The vast majority of results remained robust after considering the potentially confounding role of somatic multimorbidity and other clinical and sociodemographic factors.

**Conclusions:**

A wide range of physical illnesses are more likely to result in all-cause death in people with pre-existing severe mental disorders. This premature mortality cannot be fully explained by having more clinically recorded physical illness, suggesting that physical disorders are more likely to be fatally harmful in this patient group.

Comorbidity of mental and physical health conditions has been called ‘a key problem for medicine in the 21st century’.^1^ Studies using nationwide health registers show that people with severe mental disorders have an elevated risk of developing a large number of physical health conditions compared with people without severe mental disorders.^2–4^ This patient group is also more likely to die prematurely,^3,5–8^ with deaths from comorbid physical health conditions far outweighing the effect of suicide and accident.^5^

However, it is uncertain whether people with severe mental disorders experience premature mortality solely because they are more likely to develop a larger number of physical illnesses, or whether those illnesses are also more likely to result in death due to biological, behavioural, sociodemographic and structural factors that are related to this patient group. Substance use disorders seem to increase the fatally harmful effect of subsequent physical health conditions,^9^ but no national study of people with severe mental disorders has considered the temporal order of the mental and physical health conditions and the contribution of severe mental disorders to fatally harmful effects of physical illness.

Thus, the aim of the current study was to investigate the risk of all-cause death and loss of life-years in people with physical health conditions who had a pre-existing severe mental disorder compared with matched counterparts who had the same physical health condition but did not have a severe mental disorder. In sensitivity analyses, we considered the potentially confounding role of somatic multimorbidity as well as disorders due to psychoactive substance use, the number of past hospital admissions, and sociodemographic factors. We hypothesised that people with pre-existing severe mental disorders would have a consistently increased risk of all-cause death as well as larger losses of life-years following the onset of physical health conditions than their matched counterparts.

## Method

We performed a cohort study based on routinely collected Czech national health data, investigating all-cause mortality in individuals with pre-existing severe mental disorders compared with matched counterparts without pre-existing severe mental disorders. The research questions and analytical plan were registered at the Open Science Framework before data analyses started.^10^ Any deviations from the analytical plan are described in the supplementary material (Supplementary Methods section) available at https://doi.org/10.1192/bjp.2024.110.

### Data

We used individual-level, de-identified data from the Czech nationwide registers of all-cause hospital admissions and all-cause deaths, encompassing the period from 1 January 1994 to 31 December 2017. Linkage of registers is possible by means of a unique identifier assigned at birth. The registers are maintained by the state-funded Czech Institute of Health Information and Statistics (IHIS), which granted the Czech National Institute of Mental Health (NIMH) access to complete data. The main purpose of the registers is the monitoring of public health; however, importantly, they also serve as a claims database used by Czech insurance companies. This study was approved by the Ethics Committee of the NIMH (105/23).

The register of all-cause hospital admissions comprises records created from information routinely collected by health professionals using a standard form, following each discharge from almost all Czech in-patient healthcare settings, and includes day cases. The English translation of the form and detailed description of registers is provided elsewhere.^11^ Clinical and sociodemographic characteristics collected include the dates of admission and discharge, the primary and secondary diagnoses coded according to the ICD-10, age, gender, marital status, occupation and region of residence. However, the provision of information on marital status, occupation and region of residence is not mandatory. The register of all-cause deaths consists of information based on death certificates that are routinely completed by physicians for all deaths occurring in the Czech Republic (Czechia). The provided information included the date of death, age at death, gender, the ICD-10 cause(s) of death and, if applicable, the external cause(s) of death.

**Fig. 1:**
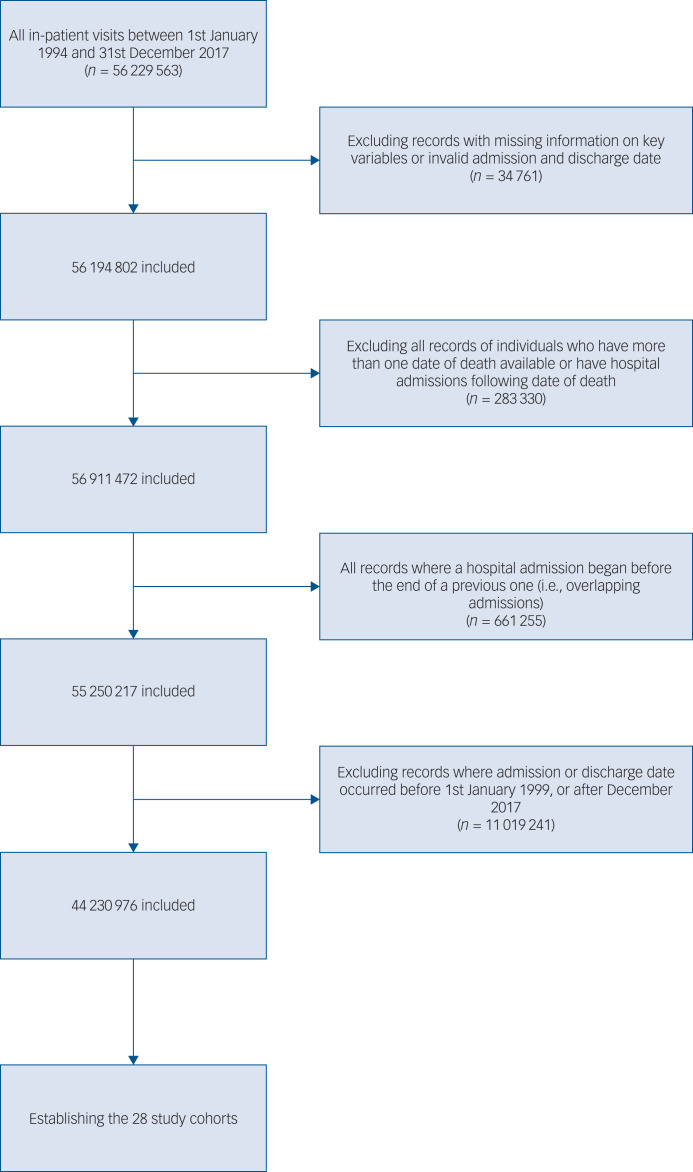
Flowchart.

We excluded (a) records with missing information on key variables (gender, age, admission and discharge date, region of residence, primary diagnosis) or incorrect (i.e. non-existent) dates, (b) all records of individuals who have more than one date of death or have hospital admissions following the date of death and (c) all records where a hospital admission began before the end of a previous one (i.e. overlapping hospital admissions). We used the first two criteria to remove records affected by administrative and/or technical errors (0.06% of all records) and the third criterion was to limit the risk of severe identification problems (negative time-to-events). For details see the flowchart in Fig. 1.

### Cohorts of people with physical health conditions

We identified all people admitted to hospital (i.e. primary diagnosis) with 1 or more of 9 broadly defined and 19 specific physical health conditions between 1 January 1999 and 31 December 2017, separately for each health condition (Supplementary Table 1). For each health condition, we considered the first occurrence as the index record (i.e. study baseline). To include incident cases of physical health conditions, we removed individuals who had a diagnosis of the specific physical health condition in the period between 1 January 1994 and the index record (i.e. wash-out period of 5 or more years) from the respective analysis. When an individual had records related to multiple physical health conditions, we included them in cohorts representing each of these physical health conditions separately (i.e. any individual could contribute more than once). We did not consider combinations of multiple different physical health conditions (e.g. cancers and diseases of the neurological system).

Then, to avoid loss to follow-up due to emigration, we excluded individuals who had region of residence listed to be outside of Czechia on their index record for a given physical health condition.

### Exposure

We defined severe mental disorders as hospital record listings of (a) psychoses (ICD-10 codes F20–F29), (b) bipolar disorder (ICD-10 code F31) or (c) severe depression (episode or recurrent; ICD-10 codes F32.2–F32.3 and F33.2–F33.3) as the primary diagnosis. We considered the occurrence of any of the above codes before the studied physical health conditions (assessed from 1 January 1999) to be indicative of having a pre-existing severe mental disorder. The comparison cohort consisted of individuals without a severe mental disorder between 1 January 1999 and the onset of the studied physical health conditions (see Supplementary Fig. 1 for an example of one condition).

### Matching

We exact-matched each individual with severe mental disorder with counterparts without severe mental disorder on the first record related to a given physical health condition on gender, age (±3 years) and discharge year listed on the record. We used matching on gender and age because we considered them as important confounders, and matching on discharge year to ensure that the individuals would have a comparable follow-up period and to control for possible calendar and cohort effects. By matching on age ±3 years, we aimed to maximise the number of matched individuals while simultaneously minimising confounding due to age. We were able to match every individual with severe mental disorder with up to five unique counterparts across all studied physical health conditions, with the exception of Parkinson's disease (2.5% unmatched individuals).

### Outcome

We investigated (a) the risk of all-cause death and (b) life-years lost following the onset of each of the 9 broadly defined and 19 specific physical health conditions.

### Statistical analysis

Following descriptive analysis of the cohorts, we used stratified Cox proportional hazards models to assess the risk of all-cause death in people with pre-existing severe mental disorders following the development of physical health conditions, when compared with counterparts without a history of severe mental disorders. Each stratum consisted of one individual with severe mental disorder and up to five individually matched, unexposed counterparts. We considered the outcome as all-cause mortality between the first record related to a given physical health condition and 31 December 2017: individuals who did not experience the outcome during the follow-up period were censored at that point. We adjusted for confounders used for matching, with age included as a continuous measure to further reduce potential residual confounding. The results are expressed as hazard ratios (HRs) with 95% confidence intervals (CIs), indicating the risk of all-cause death in people with pre-existing severe mental disorders compared with unexposed counterparts. We examined whether the proportionality assumption was fulfilled using Schoenfeld residuals. In some cases, this assumption was violated; thus, hazard ratios must be interpreted as weighted averages of the time-varying hazard ratios over the entire follow-up period.^12^ We created a Kaplan–Meier plot on all-cause death following the onset of each of the studied physical health conditions separately.

Then, we calculated differences in loss of life-years between individuals with severe mental disorders and people without severe mental disorders. We defined life-years lost as differences in remaining life expectancy^13^ after the onset of each physical health condition and before reaching the age of 81 years. We used a method that took into consideration the ages at which the physical health conditions occurred^13^ and used 10 000 bootstrap iterations to establish the 95% confidence intervals.

### Sensitivity analysis

We performed several sets of sensitivity analyses to interrogate the robustness of our results by considering different sets of potential confounders and by altering the definitions of the exposure and the outcome.

First, to account for potentially different distributions of physical health conditions in people with severe mental disorders and their counterparts that could influence the outcomes, we adjusted for the presence of other physical health conditions occurring in the 5 years prior to the index hospital admission for a given physical health condition in stratified Cox proportional hazards models, in addition to matching variables. In each broadly defined physical health condition, we adjusted for each of the 8 remaining broadly defined physical health conditions, while in each specific physical health condition, we adjusted for each of the remaining 18 physical health conditions.

Second, individuals with severe mental disorders might have a worsened overall health state relative to their counterparts, thus potentially contributing to worsened outcomes in these individuals after the onset of a given physical health condition. To partially address this, we controlled for the number of hospital admissions occurring in the 5 years prior to the index hospital admission for a given physical health condition in stratified Cox proportional hazard models, in addition to matching variables. Since the number of past hospital admissions can include admissions for severe mental disorders, and thus be part of the exposure, we also calculated this number not considering admissions for severe mental disorders.

Third, we adjusted in stratified Cox proportional hazards models for history of disorders due to psychoactive substance use (defined as ICD-10 code F1 on primary diagnosis) in the 5 years prior to the index hospital admission, in addition to matching variables. Severe mental disorders and disorders due to psychoactive substance use have a complex relationship, and we cannot rule out that for some individuals disorders due to psychoactive substance use would act as mediators, thus leading to overadjustment bias.^14^ However, we conceptually considered history of disorders due to psychoactive substance use as a confounder.

Fourth, in addition to the matching variables, we controlled for work status and marital status in the stratified Cox proportional hazards models, since these could be important confounders *per se* or proxies for socioeconomic status and social functioning. As with severe mental disorders and disorders due to psychoactive substance use, there is a possibility that work status and marital status would constitute mediators and therefore lead to overadjustment bias;^14^ however, we conceptually considered them as confounders.

Fifth, to rule out the possibility that the results might be driven by unnatural causes of death (defined as ICD-10 codes V01–Y98), we performed the analysis considering only natural causes of death as the event, with unnatural causes of death being a competing risk.

Sixth, to investigate whether severe mental disorders recorded in the distant past would be a relevant exposure, we assessed the outcome of those who had severe mental disorders recorded ≤5 years and >5 years before a given physical health condition. We note that these are subsets of the cohorts used in the main analysis.

Last, to quantify what level of confounding would be necessary to nullify the associations we observed, we computed E-values for each of our regression models where the 95% confidence intervals did not include a null effect.^15^ Higher E-values increase the confidence that the results are not due to residual confounding.^15^

Throughout the study, we followed the statement from the American Statistical Association on *P*-values; thus, we refrained from performing null-hypothesis significance testing.^16^ All analysis were performed in R (version 4.2.2 for Windows), using *survival* (version 3.5-5), *llilies* (version 0.2.129)^13^ and *EValue* (version 4.1.3).^17^

## Results

The number of individuals in disease-specific cohorts ranged from 600 (100 with and 500 without severe mental disorders) for tuberculosis to 37 962 (6327 with and 31 635 without severe mental disorders) for diseases of the circulatory system, with a median of 4593 individuals. The mean age at onset varied from around 34 years for chronic viral hepatitis to around 68 years for peripheral artery occlusive disease. The proportion of females ranged from around 28% for tuberculosis to approximately 87% for thyroid disorder (excluding prostate disorders, in which there were only males). For detailed descriptive statistics see Table 1.

**Table 1:** Descriptive statistics of the cohorts without and with severe mental disorders (SMDs)^1^

Cohort	Total, *n*		Age, years: mean (s.d.)		Females, *n* (%)		Discharge year, median (IQR)	
	Without SMDs	With SMDs	Without SMDs	With SMDs	Without SMDs	With SMDs	Without SMDs	With SMDs
Diseases of the circulatory system	31 635	6327	63.34 (12.53)	63.22 (12.58)	18 885 (59.70)	3777 (59.70)	2010 (2006–2014)	2010 (2006–2014)
Hypertension	7290	1458	62.39 (13.37)	62.27 (13.42)	5115 (70.16)	1023 (70.16)	2010 (2006–2014)	2010 (2006–2014)
Ischaemic heart disease	11 000	2200	63.92 (12.03)	63.76 (12.12)	5850 (53.18)	1170 (53.18)	2009 (2005–2013)	2009 (2005–2013)
Atrial fibrillation	3810	762	67.51 (11.20)	67.40 (11.26)	2360 (61.94)	472 (61.94)	2012 (2007–2015)	2012 (2007–2015)
Heart failure	8015	1603	67.57 (11.76)	67.29 (11.83)	5135 (64.07)	1027 (64.07)	2012 (2008–2015)	2012 (2008–2015)
Peripheral artery occlusive disease	6395	1279	68.14 (12.23)	67.96 (12.30)	3835 (59.97)	767 (59.97)	2009 (2005–2013)	2009 (2005–2013)
Stroke	11 575	2315	65.73 (12.33)	65.54 (12.36)	7000 (60.48)	1400 (60.48)	2011 (2006–2014)	2011 (2006–2014)
Diseases of the endocrine system	11 115	2223	56.90 (13.69)	56.81 (13.67)	7315 (65.81)	1463 (65.81)	2010 (2006–2014)	2010 (2006–2014)
Diabetes mellitus	8 35	1747	58.04 (13.56)	57.84 (13.56)	5245 (60.05)	1049 (60.05)	2010 (2006–2014)	2010 (2006–2014)
Thyroid disorder	2620	524	53.67 (13.32)	53.62 (13.46)	2290 (87.40)	458 (87.40)	2010 (2006–2014)	2010 (2006–2014)
Chronic pulmonary diseases	6735	1347	59.82 (14.19)	59.70 (14.17)	3985 (59.17)	797 (59.17)	2011 (2006–2014)	2011 (2006–2014)
Diseases of the gastrointestinal system	8080	1616	54.13 (16.34)	54.03 (16.31)	3945 (48.82)	789 (48.82)	2010 (2006–2014)	2010 (2006–2014)
Ulcer or chronic gastritis	3515	703	58.35 (14.75)	58.25 (14.74)	1790 (50.92)	358 (50.92)	2010 (2006–2014)	2010 (2006–2014)
Chronic liver disease	2540	508	45.10 (14.56)	44.99 (14.60)	895 (35.24)	179 (35.24)	2009 (2005–2014)	2009 (2005–2014)
Inflammatory bowel disease	760	152	46.06 (17.16)	46.07 (17.05)	380 (50.00)	76 (50.00)	2010 (2005–2014)	2010 (2005–2014)
Diverticular disease of intestine	1710	342	63.27 (12.28)	63.12 (12.33)	1115 (65.20)	223 (65.20)	2011 (2007–2014)	2011 (2007–2014)
Diseases of the urogenital system	3845	769	63.57 (11.25)	63.31 (11.34)	1165 (30.30)	233 (30.30)	2011 (2007–2014)	2011 (2007–2014)
Chronic kidney disease	1805	361	63.80 (13.86)	63.61 (13.89)	1165 (64.54)	233 (64.54)	2011 (2007–2015)	2011 (2007–2015)
Prostate disorders	2109	422	63.39 (8.43)	63.16 (8.60)	0 (0.00)	0 (0.00)	2010 (2006–2014)	2010 (2006–2014)
Connective tissue disorders	800	160	52.65 (14.52)	52.61 (14.54)	540 (67.50)	108 (67.50)	2010.5 (2006–2013)	2010.5 (2006–2013)
Cancers	18 120	3624	60.87 (11.82)	60.77 (11.87)	11 630 (64.18)	2 326 (64.18)	2011 (2007–2014)	2011 (2007–2014)
Diseases of the neurological system	9130	1826	49.79 (17.28)	49.77 (17.23)	4735 (51.86)	947 (51.86)	2010 (2006–2014)	2010 (2006–2014)
Epilepsy	6764	1353	46.85 (16.55)	46.81 (16.49)	3264 (48.26)	653 (48.26)	2010 (2006–2014)	2010 (2006–2014)
Parkinson's disease	1875	391	65.62 (11.32)	63.93 (13.04)	1081 (57.65)	225 (57.54)	2011 (2006–2014)	2011 (2006.5–2014)
Multiple sclerosis	585	117	40.87 (11.90)	41.01 (11.89)	425 (72.65)	85 (72.65)	2009 (2005–2014)	2009 (2005–2014)
Infectious and parasitic diseases	1135	227	42.69 (15.98)	42.63 (16.12)	370 (32.60)	74 (32.60)	2008 (2004–2012)	2008 (2004–2012)
Tuberculosis	500	100	53.47 (14.54)	53.34 (14.62)	140 (28.00)	28 (28.00)	2008 (2005–2012)	2008 (2005–2012)
Chronic viral hepatitis	592	119	34.03 (11.48)	34.19 (11.60)	202 (34.12)	41 (34.45)	2007 (2003–2011)	2007 (2003–2011)

IQR, interquartile range.

1 Individuals with and without severe mental disorders were exactly matched on gender, age (± 3 years) and discharge year.

### Risk of all-cause death

We detected an elevated risk of all-cause death in people with severe mental disorders following the onset of seven out of the nine studied broadly defined physical health conditions, when compared with matched counterparts. The hazard ratios for these conditions ranged from 1.20 (95% CI 1.09–1.32) for diseases of the neurological system to 1.91 (95% CI 1.83–2.00) for diseases of the circulatory system. For connective tissue disorders and infectious and parasitic diseases, the results were consistent with a null effect.

Considering specific physical health conditions, we detected an increased risk of all-cause death in people with severe mental disorders following the onset of 14 out of 19 conditions. The hazard ratios ranged from 1.24 (95% CI 1.06–1.46) for chronic kidney disease to 3.01 (95% CI 2.30–3.93) for thyroid disorder. The results for chronic liver disease, epilepsy, Parkinson's disease, tuberculosis and chronic viral hepatitis were consistent with a null effect. For detailed information see Fig. 2, Supplementary Tables 2 and 3, and Supplementary Figs 2–29.

**Fig. 2:**
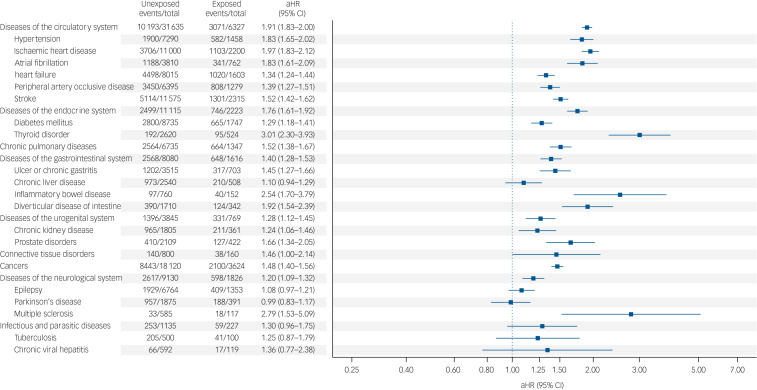
Adjusted hazard ratios (aHR) of all-cause mortality following the onset of physical health conditions in people with pre-existing severe mental disorders compared with matched counterparts without severe mental disorders. The models were adjusted for gender, age and discharge year listed on the first hospital admission for the respective physical health condition.

### Differences in losses of life-years

We detected that people with severe mental disorders had shorter life expectancy after the onset of a physical health condition than people without severe mental disorders for eight out of nine broadly defined physical health conditions. The additional losses of life-years ranged from 1.73 (95% CI 0.88–2.57) for diseases of the neurological system to 4.38 (95% CI 1.45–7.27) for connective tissue disorders. For infectious and parasitic diseases, the results were consistent with no differences in life-years lost.

**Fig. 3:**
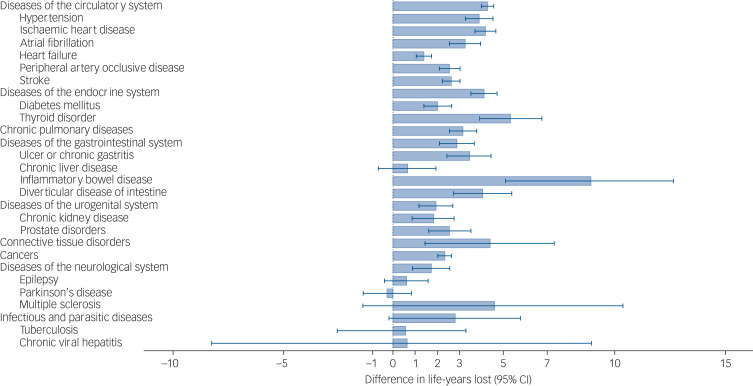
Differences in life-years lost following the onset of physical health conditions between people with pre-existing severe mental disorders and matched counterparts without severe mental disorders.

Considering specific physical health conditions, people with severe mental disorders lost more life-years following the onset of 13 out of 19 specific physical health conditions. The additional losses of life-years ranged from 1.40 (95% CI 1.05–1.74) for heart failure to 8.94 (95% CI 5.08–12.66) for inflammatory bowel disease. The results for tuberculosis, chronic viral hepatitis, Parkinson's disease, multiple sclerosis, epilepsy and chronic liver disease were consistent with no differences in life-years lost. For detailed information see Fig. 3 and Supplementary Table 4.

### Sensitivity analysis

For five out of seven broadly defined physical health conditions for which we found elevated risks in the main analysis, the results remained robust following adjustment for other physical health conditions, number of past hospital admissions, history of disorders due to psychoactive substance use and additional sociodemographic characteristics. For diseases of the urogenital system and diseases of the neurological system, the results of at least one sensitivity analysis were consistent with a null effect. Considering specific physical health conditions, we found results consistent with the main analysis for 12 out of 14 conditions. For chronic kidney disease and multiple sclerosis, the results of at least one sensitivity analysis were consistent with a null effect. See details in Table 2 and Supplementary Tables 5–7.

**Table 2:** Sensitivity analyses of all-cause mortality following the onset of physical health conditions in people with pre-existing severe mental disorders compared with matched counterparts without severe mental disorders (SMDs)^a^

Cohort	Adjusting for history of other studied physical health conditions	Adjusting for number of past hospital admissions	Adjusting for number of past hospital admissions, excluding those for severe mental disorders	Adjusting for history of disorders due to psychoactive substance use	Adjusting for additional sociodemographic characteristics	Unnatural cause of death as competing risk	SMDs ≤5 years before the physical health condition	SMDs >5 years before the physical health condition
Diseases of the circulatory system	1.86 (1.78–1.95)	1.57 (1.49–1.64)	1.75 (1.67–1.83)	1.87 (1.79–1.96)	1.66 (1.58–1.74)	1.88 (1.79–1.96)	1.91 (1.80–2.03)	1.84 (1.72–1.96)
Hypertension	1.78 (1.61–1.98)	1.54 (1.38–1.71)	1.67 (1.50–1.85)	1.80 (1.62–1.99)	1.61 (1.45–1.79)	1.77 (1.60–1.96)	1.88 (1.65–2.14)	1.77 (1.52–2.06)
Ischaemic heart disease	1.90 (1.76–2.05)	1.57 (1.45–1.71)	1.76 (1.63–1.90)	1.95 (1.81–2.10)	1.75 (1.62–1.89)	1.92 (1.78–2.07)	1.85 (1.68–2.03)	1.95 (1.73–2.19)
Atrial fibrillation	1.78 (1.56–2.04)	1.61 (1.40–1.84)	1.72 (1.50–1.97)	1.79 (1.57–2.05)	1.75 (1.53–2.01)	1.79 (1.56–2.05)	1.87 (1.55–2.25)	1.82 (1.51–2.20)
Heart failure	1.36 (1.26–1.46)	1.23 (1.14–1.33)	1.30 (1.20–1.40)	1.34 (1.24–1.44)	1.23 (1.14–1.33)	1.33 (1.23–1.43)	1.42 (1.27–1.59)	1.39 (1.26–1.54)
Peripheral artery occlusive disease	1.41 (1.29–1.54)	1.22 (1.11–1.33)	1.30 (1.19–1.42)	1.38 (1.26–1.50)	1.30 (1.19–1.41)	1.38 (1.26–1.50)	1.35 (1.20–1.51)	1.55 (1.36–1.77)
Stroke	1.52 (1.42–1.62)	1.34 (1.25–1.44)	1.44 (1.34–1.54)	1.50 (1.40–1.60)	1.39 (1.30–1.49)	1.51 (1.41–1.61)	1.58 (1.44–1.74)	1.46 (1.33–1.61)
Diseases of the endocrine system	1.76 (1.61–1.92)	1.45 (1.32–1.59)	1.63 (1.49–1.79)	1.70 (1.56–1.86)	1.47 (1.34–1.62)	1.70 (1.55–1.86)	1.60 (1.42–1.79)	1.62 (1.42–1.85)
Diabetes mellitus	1.31 (1.19–1.44)	1.11 (1.01–1.22)	1.23 (1.12–1.35)	1.26 (1.15–1.38)	1.14 (1.03–1.25)	1.26 (1.15–1.38)	1.28 (1.13–1.45)	1.30 (1.14–1.49)
Thyroid disorder	3.02 (2.27–4.01)	2.13 (1.58–2.85)	2.63 (1.99–3.46)	2.92 (2.23–3.83)	2.50 (1.89–3.32)	2.70 (2.05–3.56)	3.24 (2.33–4.50)	2.66 (1.71–4.14)
Chronic pulmonary diseases	1.52 (1.39–1.68)	1.36 (1.23–1.50)	1.44 (1.31–1.58)	1.49 (1.35–1.63)	1.33 (1.21–1.46)	1.49 (1.36–1.64)	1.54 (1.34–1.75)	1.46 (1.28–1.66)
Diseases of the gastrointestinal system	1.40 (1.28–1.54)	1.21 (1.10–1.33)	1.29 (1.18–1.42)	1.32 (1.20–1.45)	1.15 (1.04–1.26)	1.34 (1.22–1.48)	1.56 (1.37–1.77)	1.45 (1.27–1.66)
Ulcer or chronic gastritis	1.46 (1.27–1.67)	1.26 (1.10–1.45)	1.36 (1.18–1.56)	1.42 (1.24–1.62)	1.27 (1.10–1.46)	1.40 (1.22–1.60)	1.57 (1.31–1.89)	1.31 (1.08–1.58)
Chronic liver disease	1.11 (0.95–1.31)	1.00 (0.84–1.18)	1.03 (0.87–1.21)	1.05 (0.89–1.23)	0.96 (0.82–1.14)	1.07 (0.91–1.26)	0.98 (0.79–1.21)	1.12 (0.89–1.42)
Inflammatory bowel disease	2.72 (1.75–4.22)	1.85 (1.20–2.87)	2.16 (1.42–3.28)	2.50 (1.65–3.78)	2.07 (1.32–3.23)	2.22 (1.45–3.41)	2.41 (1.47–3.94)	2.83 (1.46–5.48)
Diverticular disease of intestine	2.08 (1.65–2.63)	1.75 (1.39–2.21)	1.81 (1.45–2.27)	1.91 (1.53–2.38)	1.75 (1.39–2.20)	1.79 (1.42–2.24)	2.22 (1.64–3.02)	2.05 (1.48–2.84)
Diseases of the urogenital system	1.31 (1.15–1.49)	1.06 (0.92–1.21)	1.18 (1.03–1.35)	1.26 (1.11–1.44)	1.10 (0.96–1.26)	1.24 (1.09–1.42)	1.39 (1.17–1.66)	1.29 (1.06–1.55)
Chronic kidney disease	1.24 (1.05–1.46)	1.10 (0.93–1.30)	1.15 (0.98–1.36)	1.23 (1.04–1.44)	1.17 (0.99–1.38)	1.24 (1.06–1.46)	1.26 (1.00–1.59)	1.07 (0.85–1.34)
Prostate disorders	1.68 (1.35–2.09)	1.33 (1.06–1.66)	1.52 (1.22–1.88)	1.66 (1.35–2.05)	1.40 (1.13–1.75)	1.59 (1.28–1.98)	1.68 (1.28–2.22)	2.08 (1.48–2.93)
Connective tissue disorders	1.26 (0.84–1.89)	1.02 (0.67–1.57)	1.20 (0.80–1.80)	1.48 (1.01–2.17)	1.24 (0.83–1.85)	1.35 (0.91–2.00)	1.24 (0.77–1.98)	1.70 (0.87–3.31)
Cancers	1.46 (1.39–1.54)	1.35 (1.27–1.42)	1.42 (1.35–1.50)	1.47 (1.39–1.55)	1.32 (1.25–1.39)	1.46 (1.39–1.54)	1.39 (1.29–1.51)	1.52 (1.42–1.63)
Diseases of the neurological system	1.29 (1.17–1.42)	0.99 (0.89–1.09)	1.13 (1.02–1.24)	1.16 (1.05–1.27)	1.08 (0.98–1.19)	1.16 (1.05–1.28)	1.11 (0.98–1.26)	1.23 (1.07–1.41)
Epilepsy	1.12 (1.00–1.26)	0.89 (0.79–1.01)	1.01 (0.90–1.13)	1.05 (0.94–1.18)	0.96 (0.85–1.07)	1.05 (0.93–1.18)	1.10 (0.93–1.29)	1.18 (1.00–1.38)
Parkinson's disease	0.97 (0.82–1.16)	0.91 (0.76–1.08)	0.95 (0.80–1.13)	0.98 (0.82–1.16)	0.93 (0.79–1.11)	0.99 (0.83–1.18)	0.89 (0.71–1.12)	1.18 (0.91–1.52)
Multiple sclerosis	3.17 (1.64–6.13)	2.13 (1.12–4.06)	2.67 (1.44–4.97)	2.68 (1.45–4.94)	2.84 (1.38–5.84)	2.15 (1.12–4.12)	3.48 (1.55–7.79)	1.70 (0.70–4.10)
Infectious and parasitic diseases	1.26 (0.93–1.72)	1.15 (0.83–1.58)	1.23 (0.90–1.67)	1.25 (0.92–1.70)	1.10 (0.80–1.52)	1.20 (0.87–1.66)	1.36 (0.93–1.99)	1.19 (0.72–1.96)
Tuberculosis	1.26 (0.85–1.85)	1.11 (0.76–1.63)	1.17 (0.81–1.70)	1.22 (0.84–1.77)	1.12 (0.77–1.64)	1.24 (0.86–1.80)	0.99 (0.60–1.61)	1.39 (0.82–2.37)
Chronic viral hepatitis	1.45 (0.79–2.67)	0.98 (0.52–1.86)	1.12 (0.61–2.02)	1.29 (0.71–2.34)	1.23 (0.67–2.25)	1.17 (0.58–2.37)	1.52 (0.84–2.75)	0.69 (0.14–3.33)

a. The results are expressed as hazard ratios with 95% confidence intervals. History of other studied physical health conditions, history of disorders due to psychoactive substance use and the number of past hospital admissions refer to the 5 years prior to the first hospital admission for the respective physical health condition. The additional sociodemographic characteristics were work status and marital status, both recorded at the first hospital admission for the respective physical health condition. Unnatural causes of death included suicide, accident and assault. Analyses of severe mental disorders recorded ≤5 years and >5 years before the physical health conditions are subsets of the main analysis, thus resulting in different cohort sizes and associated descriptive statistics.

The E-values for conditions that were inconsistent with a null effect in both the main and the sensitivity analysis ranged from 1.67 for diabetes mellitus to 3.67 for thyroid disorder (Supplementary Table 8).

## Discussion

### Principal findings

Using data from the Czech national register of in-patient care, we demonstrated that people with severe mental disorders were more likely to die than people without severe mental disorders following the development of 7 out of 9 broadly defined and 14 out of 19 specific physical health conditions. For most associations, particularly those related to cardiovascular diseases and cancers, the results remained robust after considering the potentially confounding role of somatic multimorbidity as well as disorders due to psychoactive substance use, the number of past hospital admissions and sociodemographic factors. Compared with people without severe mental disorders, people with pre-existing severe mental disorders showed marked additional losses of life-years in most of the studied physical health conditions. These results suggest that a wide range of physical health conditions are more likely to result in all-cause death when they occur in people with pre-existing severe mental disorders, and these associations cannot be entirely explained by this patient group having more clinically recorded physical illness.

### Comparison with other studies

To the best of our knowledge, this is the first national study to systematically investigate mortality and loss of life-years in people with severe mental disorders who subsequently develop physical health conditions. A Danish nationwide study, while not taking into consideration the temporal order of mental illness and physical health conditions, compared individuals with schizophrenia who also had physical health conditions with individuals who only had the physical health conditions, and found increased mortality and excess life-years lost in nine out of nine broadly defined physical health conditions.^18^ The magnitude of the associations we detected in our study was, on average, smaller. This might be related to differences in case mix, methodology (with our study focusing on the importance of the temporal order of severe mental disorders and physical health conditions), and the underlying populations and healthcare systems. Another Danish study based on national register data demonstrated higher risk of all-cause death in 18 out of 19 physical health conditions in individuals with pre-existing depression.^19^ The strength of the associations is broadly in line with those detected in our study; however, the authors considered many physical health conditions that we did not consider.^19^ Further contributing to limited comparability, the authors considered all occurrences of depression, including those of mild and moderate severity, and did not consider the outcomes in individuals with pre-existing depression compared with matched counterparts without pre-existing depression.^19^ When compared with our own previous study, which investigated the risk of all-cause death and loss of life-years following the development of physical health conditions in people with substance use disorders, we found that, for most conditions, people with substance use disorders displayed even higher risks of all-cause death and larger losses of life-years than people with severe mental disorders.^9^

Multiple factors might be responsible for the worsened outcomes of physical health conditions arising in people with pre-existing severe mental disorders. Suboptimal nutrition, exercise and lifestyle factors such as smoking tobacco are prevalent in this patient group.^20–22^ Antipsychotic use is associated with decreased risk of all-cause mortality in people with psychotic disorders;^23^ however, it can lead to metabolic side-effects of varying degree.^24^ There may be reluctance or difficulties, importantly, due to socioeconomic factors^25–28^ in people with severe mental disorders accessing or engaging with screening programmes,^29,30^ dental^31,32^ and surgical health services,^33^ and difficulties with adherence to treatments, including those for physical health conditions.^34^ People with severe mental disorders may experience delayed diagnosis^35,36^ or complete unrecognition of physical health conditions,^37^ potentially owing to misattribution of physical symptoms to mental disorders by medical professionals (i.e. diagnostic overshadowing).^38^ Consequently, the worsened outcomes in people with severe mental disorders may be related to higher severity of physical health conditions at their initial diagnosis and/or the presence of clinically unrecognised physical health conditions. The widespread stigma,^39^ including among medical professionals,^40^ and discrimination^41^ towards people with severe mental disorders may contribute to their lower service utilisation^42,43^ and consequently decrease the attention paid to their physical health. Finally, the healthcare system is fragmented beyond primary care, with separation between out-patient and in-patient services and between physical and mental health services,^44^ creating obstacles for people with severe mental disorders to getting their health conditions addressed in an integrated manner and militating against holistic awareness and training of clinical staff, who see themselves as managing either physical or mental disorders.

### Clinical implications

The World Health Organization emphasises the need for better physical health in people with mental disorders and calls for an integrated approach to care.^45^ Several countries have policies and national guidelines in place to improve the physical health of people with severe mental disorders. For instance, the UK National Institute for Health and Care Excellence includes physical health management in its guidance on the treatment of first-episode psychotic disorders and schizophrenia,^46^ considering that secondary mental health services should lead physical health management, certainly during the initial phase of the mental disorder. The Czech Psychiatric Association has recently issued recommendations on monitoring and addressing physical health in people with severe mental disorders. These include, among other things, the regular monitoring of biomarkers such as high-density lipoprotein, low-density lipoprotein and triglycerides.^47^ However, the existing national recommendations do not acknowledge the notion of physical disorders being more likely to be fatally harmful in this patient group. Our findings clearly demonstrate that people with severe mental disorders are particularly vulnerable and should be a high priority not only within psychiatric but also within broader health services. Ensuring the provision of holistic care for severe mental disorders and physical health conditions can be considered as a minimally adequate first step, and requires a health system-wide collaborative change. However, to fully reverse the adverse outcomes experienced by people with severe mental disorders, systemic efforts encompassing changes to public perception, policy, public health and clinical practice are required.

### Methodological considerations

Strengths of this study include the use of nationwide, routinely collected, standardised health and mortality data. This supported the analysis of usefully precise matched cohorts of people with and without severe mental disorders who developed a range of common physical health conditions. Our design lends confidence that the associations regarding increased mortality would be driven by pre-existing severe mental disorders and its consequences rather than physical illnesses leading to severe mental disorders as well as to death.

Our study has some limitations. First, the cohorts consisted of individuals treated in in-patient settings. However, a large proportion of the physical health conditions will be diagnosed and managed in community settings; thus, it could be argued that diagnoses reached following in-patient admission might be more severe and demonstrate specificity over sensitivity. This would not contribute to selection bias since all in-patient settings were considered, but it would potentially limit the generalisability of results beyond in-patient care. Second, we aimed to include only incident cases of physical health conditions, but we cannot rule out that some individuals already had these before the onset of severe mental disorder. Third, the register used in this study did not cover the entire lifespans of all included individuals, and we cannot rule out the possibility that some of the matched counterparts of people with severe mental disorders previously had severe mental disorders themselves. This would then constitute a conservative bias, resulting in underestimation of the true effects. Fourth, our data did not include information on several biological, behavioural and sociodemographic confounders, most notably body mass index, prescription medication use, smoking status and income; thus, part of our results could be due to residual confounding. Fifth, some cohorts were very small, leading to excessive uncertainty in estimates (e.g. estimates of life-years lost in connective tissue disorders). Relatedly, the size of cohorts precluded us from investigating the outcomes of people with specific severe mental disorders. Last, although the number of individuals emigrating from Czechia is low,^9^ we did not have information on emigration status, so it is possible that some individuals were lost to follow-up.

### Conclusions

Almost all categories of physical illness are more likely to result in all-cause death in people with pre-existing severe mental disorders. This premature mortality cannot be fully explained by having more clinically recorded physical illness, suggesting that the physical disorders are also more likely to be fatally harmful in this patient group. Implementing holistic care for people with severe mental disorders and physical health conditions is the necessary first step; however, coordinated changes to policy, public health and clinical practice are imperative to fully reverse the adverse outcomes experienced by this patient group.

## Supporting information

Formánek et al. supplementary material Formánek et al. supplementary material

## Data Availability

Owing to their sensitive nature, the data cannot be published or shared with external individuals without permission from the Czech Institute of Health Information and Statistics. The full analytical code of the study is available at https://github.com/tmfmnk/Severe-mental-disorders-contributing-to-mortality-following-physical-disorders. T.F. and K.M. had full access to all data in the study and take responsibility for the integrity of the data and the accuracy of the data analysis.
