# Supplementary material for: Contribution of severe mental disorders to fatally harmful effects of physical disorders: national cohort study
Source: Br J Psychiatry. 2024 Oct;225(4):436–45. doi: 10.1192/bjp.2024.110 (PMC11557285; doi:10.1192/bjp.2024.110)
Supplement: Formánek et al. supplementary material [file S0007125024001107sup001.docx]

Table of Contents

[Supplementary Methods 3](#_Toc169248113)

[Deviations from the analytical plan 3](#_Toc169248114)

[Supplementary Results 5](#_Toc169248115)

[Supplementary Table 1 ICD-10 diagnosis codes of considered physical health conditions 5](#_Toc169248116)

[Supplementary Table 2 Stratified Cox proportional hazards models of all-cause mortality following the onset of physical health conditions in people with severe mental disorder 6](#_Toc169248117)

[Supplementary Table 3 Absolute risks of all-cause mortality following the onset of physical health conditions in people with and without severe mental disorder 7](#_Toc169248118)

[Supplementary Table 4 Differences in life-years lost following the onset of physical health conditions between people with severe mental disorder and people without severe mental disorder 9](#_Toc169248119)

[Supplementary Table 5 Natural and unnatural causes of death in people with severe mental disorder and their matched counterparts 10](#_Toc169248120)

[Supplementary Table 6 Descriptive statistics of cohorts, severe mental disorder recorded 5<= years before the physical health condition 11](#_Toc169248121)

[Supplementary Table 7 Descriptive statistics of cohorts, severe mental disorder recorded >5 years before the physical health condition 13](#_Toc169248122)

[Supplementary Table 8 E-values 15](#_Toc169248123)

[Supplementary Figure 1 Study design, example of pre-existing severe mental disorder and subsequent diabetes mellitus 16](#_Toc169248124)

[Supplementary Figure 2 Survival probability with 95% confidence interval following the first hospitalization for atrial fibrillation in people with and without severe mental disorder 17](#_Toc169248125)

[Supplementary Figure 3 Survival probability with 95% confidence interval following the first hospitalization for cancers in people with and without severe mental disorder 18](#_Toc169248126)

[Supplementary Figure 4 Survival probability with 95% confidence interval following the first hospitalization for chronic kidney disease in people with and without severe mental disorder 19](#_Toc169248127)

[Supplementary Figure 5 Survival probability with 95% confidence interval following the first hospitalization for chronic liver disease in people with and without severe mental disorder 20](#_Toc169248128)

[Supplementary Figure 6 Survival probability with 95% confidence interval following the first hospitalization for chronic pulmonary diseases in people with and without severe mental disorder 21](#_Toc169248129)

[Supplementary Figure 7 Survival probability with 95% confidence interval following the first hospitalization for chronic viral hepatitis in people with and without severe mental disorder 22](#_Toc169248130)

[Supplementary Figure 8 Survival probability with 95% confidence interval following the first hospitalization for connective tissue disorders in people with and without severe mental disorder 23](#_Toc169248131)

[Supplementary Figure 9 Survival probability with 95% confidence interval following the first hospitalization for diabetes mellitus in people with and without severe mental disorder 24](#_Toc169248132)

[Supplementary Figure 10 Survival probability with 95% confidence interval following the first hospitalization for diseases of the circulatory system in people with and without severe mental disorder 25](#_Toc169248133)

[Supplementary Figure 11 Survival probability with 95% confidence interval following the first hospitalization for diseases of the endocrine system in people with and without severe mental disorder 26](#_Toc169248134)

[Supplementary Figure 12 Survival probability with 95% confidence interval following the first hospitalization for diseases of the gastrointestinal system in people with and without severe mental disorder 27](#_Toc169248135)

[Supplementary Figure 13 Survival probability with 95% confidence interval following the first hospitalization for diseases of the neurological system in people with and without severe mental disorder 28](#_Toc169248136)

[Supplementary Figure 14 Survival probability with 95% confidence interval following the first hospitalization for diseases of the urogenital system in people with and without severe mental disorder 29](#_Toc169248137)

[Supplementary Figure 15 Survival probability with 95% confidence interval following the first hospitalization for diverticular disease of the intestine in people with and without severe mental disorder 30](#_Toc169248138)

[Supplementary Figure 16 Survival probability with 95% confidence interval following the first hospitalization for epilepsy in people with and without severe mental disorder 31](#_Toc169248139)

[Supplementary Figure 17 Survival probability with 95% confidence interval following the first hospitalization for heart failure in people with and without severe mental disorder 32](#_Toc169248140)

[Supplementary Figure 18 Survival probability with 95% confidence interval following the first hospitalization for hypertension in people with and without severe mental disorder 33](#_Toc169248141)

[Supplementary Figure 19 Survival probability with 95% confidence interval following the first hospitalization for infectious and parasitic diseases in people with and without severe mental disorder 34](#_Toc169248142)

[Supplementary Figure 20 Survival probability with 95% confidence interval following the first hospitalization for inflammatory bowel disease in people with and without severe mental disorder 35](#_Toc169248143)

[Supplementary Figure 21 Survival probability with 95% confidence interval following the first hospitalization for ischemic heart disease in people with and without severe mental disorder 36](#_Toc169248144)

[Supplementary Figure 22 Survival probability with 95% confidence interval following the first hospitalization for multiple sclerosis in people with and without severe mental disorder 37](#_Toc169248145)

[Supplementary Figure 23 Survival probability with 95% confidence interval following the first hospitalization for Parkinson’ s disease in people with and without severe mental disorder 38](#_Toc169248146)

[Supplementary Figure 24 Survival probability with 95% confidence interval following the first hospitalization for peripheral artery occlusive disease in people with and without severe mental disorder 39](#_Toc169248147)

[Supplementary Figure 25 Survival probability with 95% confidence interval following the first hospitalization for prostate disorders in people with and without severe mental disorder 40](#_Toc169248148)

[Supplementary Figure 26 Survival probability with 95% confidence interval following the first hospitalization for stroke in people with and without severe mental disorder 41](#_Toc169248149)

[Supplementary Figure 27 Survival probability with 95% confidence interval following the first hospitalization for thyroid disorder in people with and without severe mental disorder 42](#_Toc169248150)

[Supplementary Figure 28 Survival probability with 95% confidence interval following the first hospitalization for tuberculosis in people with and without severe mental disorder 43](#_Toc169248151)

[Supplementary Figure 29 Survival probability with 95% confidence interval following the first hospitalization for ulcer or chronic gastritis in people with and without severe mental disorder 44](#_Toc169248152)

[References 45](#_Toc169248153)

# Supplementary Methods

## Deviations from the analytical plan

The research questions and the analytical plan were pre-registered at Open Science Framework before data analyses started (1). We list below the deviations from the analytical plan.

First, we considered major depressive episode using ICD-10 codes F32-F33 as severe mental disorder. However, the ICD-10 codes F320 and F321 refer to mild and moderate depressive episode, while the ICD-10 codes F330 and F331 refer to mild and moderate recurrent depressive disorder. To ensure that the exposure consistently captures severe mental disorder, we restricted the analysis to ICD-10 codes F322, F323, F332, and F333, referring to severe depressive episode (with and without psychotic symptoms) and severe recurrent depressive disorders (with and without psychotic symptoms). In addition to this, from psychoses, we included schizophrenia (ICD-10 code F20) and schizoaffective disorder (ICD-10 code F25). While these diseases are consistently considered as severe mental disorder, most other psychoses are also routinely considered as such. Thus, we decided to include all psychoses using ICD-10 codes F20-F29.

Second, instead of calculating the losses of life-years in people with and without severe mental disorder relative to the general population, we calculated the differences in life-years lost between people with severe mental disorder and their matched counterparts without severe mental disorder. We did so in order to provide direct comparisons between the groups. Relatedly, to increase tractability, we decided to calculate the losses of life-years across all onset ages, and before reaching the age of 81 years.

Third, we removed the age restrictions and included all individuals with the studied physical health conditions regardless of their onset age. We previously used the age interval of 15 to 70 years in the context of identifying individuals with substance use disorders to avoid records related to acute intoxications being classified as substance use disorders in younger individuals and to reflect that people with substance use disorders have a substantially reduced likelihood of living beyond the age of 70 years (2). However, in this study, we believe that none of these can be justified.

Fourth, in addition to our sensitivity analyses adjusting for the number of hospitalizations occurring in the period of five-years prior to the index hospitalization for a given physical health condition, we also fitted a model with the number of hospitalizations occurring in the period of five-years prior to the index hospitalization that excludes hospitalizations for severe mental disorder. We did so since the number of hospitalizations related to severe mental disorder might be part of the exposure (i.e., having a severe mental disorder).

Fifth, to increase the confidence that the results we detected are not due to a higher likelihood of deaths due to unnatural causes (defined as ICD-10 codes V01-Y98) in people with severe mental disorder, we performed an additional sensitivity analysis where we considered unnatural causes of death as a competing risk event.

Sixth, since the number of individuals with severe mental disorder and physical health conditions was relatively small, we were able to exactly-match them with up to five counterparts without severe mental disorder who had the same physical health condition.

Last, following peer review, we included another set of sensitivity analyses to investigate whether severe mental disorder recorded in the distant past would be a relevant exposure. To do so, we assessed the outcome of those who had severe mental disorder recorded <= 5 years and >5 years before a given physical health condition, respectively.

# Supplementary Results

## Supplementary Table 1 ICD-10 diagnosis codes of considered physical health conditions

| Subsequent physical health condition | ICD-10 codes |
| --- | --- |
| Diseases of the circulatory system | |
| Hypertension | I10-I13, I15 |
| Ischemic heart disease | I20-I25 |
| Atrial fibrillation | I48 |
| Heart failure | I50 |
| Peripheral artery occlusive disease | I70-I74 |
| Stroke | I60-I64, I69 |
| Diseases of the endocrine system | |
| Diabetes mellitus | E10-E14 |
| Thyroid disorder | E00-E05, E061-E069, E07 |
| Chronic pulmonary diseases | J40-J47 |
| Diseases of the gastrointestinal system |  |
| Ulcer or chronic gastritis | K221, K25-K28, K293-K295 |
| Chronic liver disease | B16-B19, K70, K74, K766, I85 |
| Inflammatory bowel disease | K50-K51 |
| Diverticular disease of intestine | K57 |
| Diseases of the urogenital system | |
| Chronic kidney disease | N03, N11, N18-N19 |
| Prostate disorders | N40 |
| Connective tissue disorders | M05-M06, M08-M09, M30-M36, D86 |
| Cancers | C00-C43, C45-C97 |
| Diseases of the neurological system | |
| Epilepsy | G40-G41 |
| Parkinson's disease | G20-G22 |
| Multiple sclerosis | G35 |
| Infectious and parasitic diseases | B20-B24, A15-A17, B18 |
| Tuberculosis | A15-A17 |
| Chronic viral hepatitis | B18 |

## Supplementary Table 2 Stratified Cox proportional hazards models of all-cause mortality following the onset of physical health conditions in people with severe mental disorder

| Cohort | HR (95% CI) |
| --- | --- |
| Diseases of the circulatory system | 1.91 (1.83; 2.00) |
| Hypertension | 1.83 (1.65; 2.02) |
| Ischemic heart disease | 1.97 (1.83; 2.12) |
| Atrial fibrillation | 1.83 (1.61; 2.09) |
| Heart failure | 1.34 (1.24; 1.44) |
| Peripheral artery occlusive disease | 1.39 (1.27; 1.51) |
| Stroke | 1.52 (1.42; 1.62) |
| Diseases of the endocrine system | 1.76 (1.61; 1.92) |
| Diabetes mellitus | 1.29 (1.18; 1.41) |
| Thyroid disorder | 3.01 (2.30; 3.93) |
| Chronic pulmonary diseases | 1.52 (1.38; 1.67) |
| Diseases of the gastrointestinal system | 1.40 (1.28; 1.53) |
| Ulcer or chronic gastritis | 1.45 (1.27; 1.66) |
| Chronic liver disease | 1.10 (0.94; 1.29) |
| Inflammatory bowel disease | 2.54 (1.70; 3.79) |
| Diverticular disease of intestine | 1.92 (1.54; 2.39) |
| Diseases of the urogenital system | 1.28 (1.12; 1.45) |
| Chronic kidney disease | 1.24 (1.06; 1.46) |
| Prostate disorders | 1.66 (1.34; 2.05) |
| Connective tissue disorders | 1.46 (1.00; 2.14) |
| Cancers | 1.48 (1.40; 1.56) |
| Diseases of the neurological system | 1.20 (1.09; 1.32) |
| Epilepsy | 1.08 (0.97; 1.21) |
| Parkinson's disease | 0.99 (0.83; 1.17) |
| Multiple sclerosis | 2.79 (1.53; 5.09) |
| Infectious and parasitic diseases | 1.30 (0.96; 1.75) |
| Tuberculosis | 1.25 (0.87; 1.79) |
| Chronic viral hepatitis | 1.36 (0.77; 2.38) |

## Supplementary Table 3 Absolute risks of all-cause mortality following the onset of physical health conditions in people with and without severe mental disorder

| Cohort | People without severe mental disorder,  n (%) | People with severe mental disorder,  n (%) |
| --- | --- | --- |
| Diseases of the circulatory system | 10 193 (32.22) | 3 071 (48.54) |
| Hypertension | 1 900 (26.06) | 582 (39.92) |
| Ischemic heart disease | 3 706 (33.69) | 1 103 (50.14) |
| Atrial fibrillation | 1 188 (31.18) | 341 (44.75) |
| Heart failure | 4 498 (56.12) | 1 020 (63.63) |
| Peripheral artery occlusive disease | 3 450 (53.95) | 808 (63.17) |
| Stroke | 5 114 (44.18) | 1 301 (56.20) |
| Diseases of the endocrine system | 2 499 (22.48) | 746 (33.56) |
| Diabetes mellitus | 2 800 (32.05) | 665 (38.07) |
| Thyroid disorder | 192 (7.33) | 95 (18.13) |
| Chronic pulmonary diseases | 2 564 (38.07) | 664 (49.29) |
| Diseases of the gastrointestinal system | 2 568 (31.78) | 648 (40.10) |
| Ulcer or chronic gastritis | 1 202 (34.20) | 317 (45.09) |
| Chronic liver disease | 973 (38.31) | 210 (41.34) |
| Inflammatory bowel disease | 97 (12.76) | 40 (26.32) |
| Diverticular disease of intestine | 390 (22.81) | 124 (36.26) |
| Diseases of the urogenital system | 1 396 (36.31) | 331 (43.04) |
| Chronic kidney disease | 965 (53.46) | 211 (58.45) |
| Prostate disorders | 410 (19.44) | 127 (30.09) |
| Connective tissue disorders | 140 (17.50) | 38 (23.75) |
| Cancers | 8 443 (46.59) | 2 100 (57.95) |
| Diseases of the neurological system | 2 617 (28.66) | 598 (32.75) |
| Epilepsy | 1 929 (28.52) | 409 (30.23) |
| Parkinson's disease | 957 (51.04) | 188 (48.08) |
| Multiple sclerosis | 33 (5.64) | 18 (15.38) |
| Infectious and parasitic diseases | 253 (22.29) | 59 (25.99) |
| Tuberculosis | 205 (41.00) | 41 (41.00) |
| Chronic viral hepatitis | 66 (11.15) | 17 (14.29) |

## Supplementary Table 4 Differences in life-years lost following the onset of physical health conditions between people with severe mental disorder and people without severe mental disorder

| Cohort | Difference in life-years lost (95% CI) |
| --- | --- |
| Diseases of the circulatory system | 4.27 (3.99; 4.55) |
| Hypertension | 3.89 (3.27; 4.51) |
| Ischemic heart disease | 4.17 (3.70; 4.64) |
| Atrial fibrillation | 3.26 (2.55; 3.96) |
| Heart failure | 1.40 (1.05; 1.74) |
| Peripheral artery occlusive disease | 2.55 (2.09; 3.03) |
| Stroke | 2.63 (2.24; 3.02) |
| Diseases of the endocrine system | 4.11 (3.52; 4.70) |
| Diabetes mellitus | 2.01 (1.40; 2.65) |
| Thyroid disorder | 5.30 (3.91; 6.72) |
| Chronic pulmonary diseases | 3.16 (2.55; 3.78) |
| Diseases of the gastrointestinal system | 2.88 (2.10; 3.67) |
| Ulcer or chronic gastritis | 3.46 (2.44; 4.43) |
| Chronic liver disease | 0.66 (-0.66; 1.94) |
| Inflammatory bowel disease | 8.94 (5.08; 12.66) |
| Diverticular disease of intestine | 4.05 (2.74; 5.36) |
| Diseases of the urogenital system | 1.94 (1.17; 2.70) |
| Chronic kidney disease | 1.84 (0.86; 2.76) |
| Prostate disorders | 2.55 (1.61; 3.52) |
| Connective tissue disorders | 4.38 (1.45; 7.27) |
| Cancers | 2.34 (2.02; 2.65) |
| Diseases of the neurological system | 1.73 (0.88; 2.57) |
| Epilepsy | 0.62 (-0.39; 1.59) |
| Parkinson's disease | -0.27 (-1.34; 0.83) |
| Multiple sclerosis | 4.58 (-1.36; 10.38) |
| Infectious and parasitic diseases | 2.81 (-0.18; 5.76) |
| Tuberculosis | 0.57 (-2.52; 3.29) |
| Chronic viral hepatitis | 0.63 (-8.18; 8.96) |

## Supplementary Table 5 Natural and unnatural causes of death in people with severe mental disorder and their matched counterparts

| Cohort | People without severe mental disorder | | People with severe mental disorder | |
| --- | --- | --- | --- | --- |
|  | Natural causes of deaths,  % | Unnatural causes of deaths,  % | Natural causes of deaths,  % | Unnatural causes of deaths,  % |
| Diseases of the circulatory system | 98.27 | 1.73 | 96.71 | 3.29 |
| Hypertension | 98.11 | 1.89 | 95.02 | 4.98 |
| Ischemic heart disease | 98.46 | 1.54 | 96.74 | 3.26 |
| Atrial fibrillation | 97.47 | 2.53 | 96.19 | 3.81 |
| Heart failure | 98.87 | 1.13 | 97.94 | 2.06 |
| Peripheral artery occlusive disease | 98.75 | 1.25 | 97.52 | 2.48 |
| Stroke | 98.79 | 1.21 | 97.85 | 2.15 |
| Diseases of the endocrine system | 98.00 | 2.00 | 95.17 | 4.83 |
| Diabetes mellitus | 98.21 | 1.79 | 95.94 | 4.06 |
| Thyroid disorder | 97.92 | 2.08 | 89.47 | 10.53 |
| Chronic pulmonary diseases | 98.09 | 1.91 | 96.39 | 3.61 |
| Diseases of the gastrointestinal system | 97.16 | 2.84 | 93.21 | 6.79 |
| Ulcer or chronic gastritis | 97.75 | 2.25 | 94.32 | 5.68 |
| Chronic liver disease | 96.20 | 3.80 | 93.81 | 6.19 |
| Inflammatory bowel disease | 97.94 | 2.06 | 85.00 | 15.00 |
| Diverticular disease of intestine | 97.18 | 2.82 | 91.13 | 8.87 |
| Diseases of the urogenital system | 98.57 | 1.43 | 96.37 | 3.63 |
| Chronic kidney disease | 98.24 | 1.76 | 98.58 | 1.42 |
| Prostate disorders | 97.07 | 2.93 | 92.91 | 7.09 |
| Connective tissue disorders | 99.29 | 0.71 | 92.11 | 7.89 |
| Cancers | 99.37 | 0.63 | 98.29 | 1.71 |
| Diseases of the neurological system | 95.53 | 4.47 | 92.47 | 7.53 |
| Epilepsy | 94.09 | 5.91 | 91.20 | 8.80 |
| Parkinson's disease | 97.60 | 2.40 | 96.81 | 3.19 |
| Multiple sclerosis | 100.00 | 0.00 | 77.78 | 22.22 |
| Infectious and parasitic diseases | 93.28 | 6.72 | 86.44 | 13.56 |
| Tuberculosis | 95.61 | 4.39 | 95.12 | 4.88 |
| Chronic viral hepatitis | 75.76 | 24.24 | 64.71 | 35.29 |

## Supplementary Table 6 Descriptive statistics of cohorts, severe mental disorder recorded 5<= years before the physical health condition

| Cohort | Total,  n | | Age,  mean (SD) | | Females,  n (%) | | Discharge year,  median (IQR) | |
| --- | --- | --- | --- | --- | --- | --- | --- | --- |
|  | People without severe mental disorder | People with severe mental disorder | People without severe mental disorder | People with severe mental disorder | People without severe mental disorder | People with severe mental disorder | People without severe mental disorder | People with severe mental disorder |
| Diseases of the circulatory system | 14 495 | 2 899 | 62.71 (13.44) | 62.55 (13.52) | 8 660 (59.74) | 1 732 (59.74) | 2006 (2003-2011) | 2006 (2003-2011) |
| Hypertension | 3 470 | 694 | 61.07 (14.58) | 60.95 (14.65) | 2 365 (68.16) | 473 (68.16) | 2006 (2003-2010) | 2006 (2003-2010) |
| Ischemic heart disease | 5 445 | 1 089 | 63.83 (12.63) | 63.70 (12.73) | 3 040 (55.83) | 608 (55.83) | 2005 (2003-2009) | 2005 (2003-2009) |
| Atrial fibrillation | 1 625 | 325 | 66.40 (12.19) | 66.32 (12.26) | 1 005 (61.85) | 201 (61.85) | 2008 (2004-2012) | 2008 (2004-2012) |
| Heart failure | 3 055 | 611 | 67.34 (12.29) | 67.02 (12.36) | 1 940 (63.50) | 388 (63.50) | 2009 (2005-2013) | 2009 (2005-2013) |
| Peripheral artery occlusive disease | 3 195 | 639 | 68.86 (12.86) | 68.70 (12.95) | 2 035 (63.69) | 407 (63.69) | 2006 (2003-2010) | 2006 (2003-2010) |
| Stroke | 5 070 | 1 014 | 65.23 (13.22) | 65.03 (13.24) | 3 050 (60.16) | 610 (60.16) | 2007 (2003-2012) | 2007 (2003-2012) |
| Diseases of the endocrine system | 5 005 | 1 001 | 56.07 (14.56) | 55.96 (14.61) | 3 360 (67.13) | 672 (67.13) | 2006 (2003-2011) | 2006 (2003-2011) |
| Diabetes mellitus | 3 800 | 760 | 57.44 (14.35) | 57.32 (14.41) | 2 335 (61.45) | 467 (61.45) | 2006 (2003-2011) | 2006 (2003-2011) |
| Thyroid disorder | 1 300 | 260 | 52.16 (14.48) | 52.10 (14.59) | 1 120 (86.15) | 224 (86.15) | 2007 (2004-2011) | 2007 (2004-2011) |
| Chronic pulmonary diseases | 2 945 | 589 | 57.98 (15.62) | 57.92 (15.54) | 1 675 (56.88) | 335 (56.88) | 2006 (2003-2011) | 2006 (2003-2011) |
| Diseases of the gastrointestinal system | 3 905 | 781 | 51.90 (17.34) | 51.79 (17.30) | 1 905 (48.78) | 381 (48.78) | 2006 (2003-2011) | 2006 (2003-2011) |
| Ulcer or chronic gastritis | 1 595 | 319 | 57.82 (15.40) | 57.81 (15.33) | 855 (53.61) | 171 (53.61) | 2006 (2003-2011) | 2006 (2003-2011) |
| Chronic liver disease | 1 410 | 282 | 42.09 (15.19) | 41.98 (15.18) | 505 (35.82) | 101 (35.82) | 2005 (2003-2010) | 2005 (2003-2010) |
| Inflammatory bowel disease | 400 | 80 | 43.73 (16.96) | 43.58 (16.99) | 205 (51.25) | 41 (51.25) | 2007 (2004-2012) | 2007 (2004-2012) |
| Diverticular disease of intestine | 740 | 148 | 63.22 (11.69) | 63.05 (11.75) | 460 (62.16) | 92 (62.16) | 2008 (2004-2012) | 2008 (2004-2012) |
| Diseases of the urogenital system | 1 795 | 359 | 63.13 (11.68) | 62.90 (11.79) | 475 (26.46) | 95 (26.46) | 2007 (2004-2012) | 2007 (2004-2012) |
| Chronic kidney disease | 775 | 155 | 63.22 (14.46) | 63.10 (14.60) | 475 (61.29) | 95 (61.29) | 2008 (2004-2012) | 2008 (2004-2012) |
| Prostate disorders | 1 059 | 212 | 63.15 (8.88) | 62.88 (9.22) | 0 (0.00) | 0 (0.00) | 2007 (2004-2012) | 2007 (2004-2012) |
| Connective tissue disorders | 440 | 88 | 52.41 (14.57) | 52.48 (14.61) | 310 (70.45) | 62 (70.45) | 2007 (2004-2012) | 2007 (2004-2012) |
| Cancers | 7 275 | 1 455 | 59.89 (12.58) | 59.78 (12.66) | 4 620 (63.51) | 924 (63.51) | 2007 (2003-2011) | 2007 (2003-2011) |
| Diseases of the neurological system | 4 880 | 976 | 46.10 (17.94) | 46.09 (17.84) | 2 530 (51.84) | 506 (51.84) | 2007 (2003.75-2012) | 2007 (2003.75-2012) |
| Epilepsy | 3 544 | 709 | 42.00 (16.47) | 42.07 (16.42) | 1 709 (48.22) | 342 (48.24) | 2007 (2003-2012) | 2007 (2003-2012) |
| Parkinson's disease | 1 009 | 211 | 64.20 (11.93) | 62.44 (13.91) | 582 (57.68) | 121 (57.35) | 2008 (2004-2013) | 2008 (2004-2013) |
| Multiple sclerosis | 305 | 61 | 38.58 (11.51) | 38.69 (11.53) | 225 (73.77) | 45 (73.77) | 2005 (2004-2011) | 2005 (2004-2011) |
| Infectious and parasitic diseases | 660 | 132 | 40.54 (16.68) | 40.51 (16.74) | 240 (36.36) | 48 (36.36) | 2005 (2003-2008) | 2005 (2003-2008) |
| Tuberculosis | 250 | 50 | 52.27 (15.84) | 52.26 (16.05) | 75 (30.00) | 15 (30.00) | 2005 (2003-2009) | 2005 (2003-2008.75) |
| Chronic viral hepatitis | 395 | 79 | 33.01 (12.29) | 33.14 (12.38) | 155 (39.24) | 31 (39.24) | 2005 (2002-2008) | 2005 (2002-2008) |

## Supplementary Table 7 Descriptive statistics of cohorts, severe mental disorder recorded >5 years before the physical health condition

| Cohort | Total,  n | | Age,  mean (SD) | | Females,  n (%) | | Discharge year,  median (IQR) | |
| --- | --- | --- | --- | --- | --- | --- | --- | --- |
|  | People without severe mental disorder | People with severe mental disorder | People without severe mental disorder | People with severe mental disorder | People without severe mental disorder | People with severe mental disorder | People without severe mental disorder | People with severe mental disorder |
| Diseases of the circulatory system | 17 140 | 3 428 | 63.95 (11.70) | 63.80 (11.71) | 10 225 (59.66) | 2 045 (59.66) | 2013 (2010-2015) | 2013 (2010-2015) |
| Hypertension | 3 820 | 764 | 63.60 (12.06) | 63.47 (12.09) | 2 750 (71.99) | 550 (71.99) | 2012 (2009-2015) | 2012 (2009-2015) |
| Ischemic heart disease | 5 555 | 1 111 | 63.94 (11.42) | 63.83 (11.50) | 2 810 (50.59) | 562 (50.59) | 2012 (2009-2015) | 2012 (2009-2015) |
| Atrial fibrillation | 2 185 | 437 | 68.36 (10.25) | 68.20 (10.41) | 1 355 (62.01) | 271 (62.01) | 2013 (2011-2016) | 2013 (2011-2016) |
| Heart failure | 4 960 | 992 | 67.71 (11.45) | 67.45 (11.50) | 3 195 (64.42) | 639 (64.42) | 2013 (2011-2016) | 2013 (2011-2016) |
| Peripheral artery occlusive disease | 3 200 | 640 | 67.33 (11.46) | 67.21 (11.58) | 1 800 (56.25) | 360 (56.25) | 2012 (2009-2015) | 2012 (2009-2015) |
| Stroke | 6 505 | 1 301 | 66.14 (11.59) | 65.94 (11.63) | 3 950 (60.72) | 790 (60.72) | 2013 (2010-2015) | 2013 (2010-2015) |
| Diseases of the endocrine system | 6 110 | 1 222 | 57.60 (12.85) | 57.50 (12.82) | 3 955 (64.73) | 791 (64.73) | 2012 (2009-2015) | 2012 (2009-2015) |
| Diabetes mellitus | 4 935 | 987 | 58.44 (12.88) | 58.24 (12.85) | 2 910 (58.97) | 582 (58.97) | 2012 (2009-2015) | 2012 (2009-2015) |
| Thyroid disorder | 1 320 | 264 | 55.06 (12.09) | 55.11 (12.09) | 1 170 (88.64) | 234 (88.64) | 2013 (2009.75-2015) | 2013 (2009.75-2015) |
| Chronic pulmonary diseases | 3 790 | 758 | 61.22 (12.94) | 61.08 (12.85) | 2 310 (60.95) | 462 (60.95) | 2013 (2010-2015) | 2013 (2010-2015) |
| Diseases of the gastrointestinal system | 4 175 | 835 | 56.19 (15.12) | 56.14 (15.03) | 2 040 (48.86) | 408 (48.86) | 2013 (2009-2015) | 2013 (2009-2015) |
| Ulcer or chronic gastritis | 1 920 | 384 | 58.71 (14.34) | 58.61 (14.24) | 935 (48.70) | 187 (48.70) | 2012 (2009-2015) | 2012 (2009-2015) |
| Chronic liver disease | 1 130 | 226 | 48.87 (12.98) | 48.75 (12.92) | 390 (34.51) | 78 (34.51) | 2013 (2010-2015) | 2013 (2010-2015) |
| Inflammatory bowel disease | 360 | 72 | 48.81 (17.08) | 48.85 (16.81) | 175 (48.61) | 35 (48.61) | 2012.5 (2009.75-2015) | 2012.5 (2009.75-2015) |
| Diverticular disease of intestine | 970 | 194 | 63.26 (12.69) | 63.16 (12.78) | 655 (67.53) | 131 (67.53) | 2013 (2010-2015) | 2013 (2010-2015) |
| Diseases of the urogenital system | 2 050 | 410 | 63.91 (10.87) | 63.66 (10.92) | 690 (33.66) | 138 (33.66) | 2013 (2010-2015) | 2013 (2010-2015) |
| Chronic kidney disease | 1 030 | 206 | 64.19 (13.42) | 64.00 (13.35) | 690 (66.99) | 138 (66.99) | 2013 (2010-2016) | 2013 (2010-2016) |
| Prostate disorders | 1 050 | 210 | 63.69 (7.78) | 63.45 (7.94) | 0 (0.00) | 0 (0.00) | 2013 (2009-2015) | 2013 (2009-2015) |
| Connective tissue disorders | 360 | 72 | 52.81 (14.53) | 52.76 (14.55) | 230 (63.89) | 46 (63.89) | 2012.5 (2010-2015.25) | 2012.5 (2010-2015.25) |
| Cancers | 10 845 | 2 169 | 61.53 (11.21) | 61.43 (11.26) | 7 010 (64.64) | 1 402 (64.64) | 2013 (2010-2015) | 2013 (2010-2015) |
| Diseases of the neurological system | 4 250 | 850 | 54.03 (15.57) | 53.99 (15.46) | 2 205 (51.88) | 441 (51.88) | 2013 (2009-2015) | 2013 (2009.25-2015) |
| Epilepsy | 3 220 | 644 | 52.08 (15.01) | 52.04 (14.92) | 1 555 (48.29) | 311 (48.29) | 2013 (2009-2015) | 2013 (2009-2015) |
| Parkinson's disease | 875 | 181 | 67.06 (10.56) | 65.50 (11.95) | 505 (57.71) | 105 (58.01) | 2013 (2010-2015) | 2013 (2010-2015) |
| Multiple sclerosis | 280 | 56 | 43.48 (11.81) | 43.54 (11.87) | 200 (71.43) | 40 (71.43) | 2012 (2008-2015) | 2012 (2008-2015) |
| Infectious and parasitic diseases | 475 | 95 | 45.73 (14.63) | 45.57 (14.79) | 130 (27.37) | 26 (27.37) | 2011 (2009-2015) | 2011 (2009-2015) |
| Tuberculosis | 250 | 50 | 54.32 (12.85) | 54.42 (13.11) | 65 (26.00) | 13 (26.00) | 2011 (2008-2015) | 2011 (2008-2015) |
| Chronic viral hepatitis | 198 | 40 | 36.36 (9.36) | 36.27 (9.70) | 48 (24.24) | 10 (25.00) | 2012 (2010-2015) | 2012 (2010-2015) |

## Supplementary Table 8 E-values

| Cohort | E-value |
| --- | --- |
| Diseases of the circulatory system | 2.50 |
| Hypertension | 2.40 |
| Ischemic heart disease | 2.57 |
| Atrial fibrillation | 2.41 |
| Heart failure | 1.75 |
| Peripheral artery occlusive disease | 1.82 |
| Stroke | 2.01 |
| Diseases of the endocrine system | 2.31 |
| Diabetes mellitus | 1.67 |
| Thyroid disorder | 3.67 |
| Chronic pulmonary diseases | 2.01 |
| Diseases of the gastrointestinal system | 1.84 |
| Ulcer or chronic gastritis | 1.91 |
| Chronic liver disease | NA |
| Inflammatory bowel disease | 3.20 |
| Diverticular disease of intestine | 2.51 |
| Diseases of the urogenital system | 1.65 |
| Chronic kidney disease | 1.60 |
| Prostate disorders | 2.19 |
| Connective tissue disorders | 1.93 |
| Cancers | 1.95 |
| Diseases of the neurological system | 1.53 |
| Epilepsy | NA |
| Parkinson's disease | NA |
| Multiple sclerosis | 3.46 |
| Infectious and parasitic diseases | NA |
| Tuberculosis | NA |
| Chronic viral hepatitis | NA |

We calculated E-values only for models that were not consistent with a null effect.

## Supplementary Figure 1 Study design, example of pre-existing severe mental disorder and subsequent diabetes mellitus


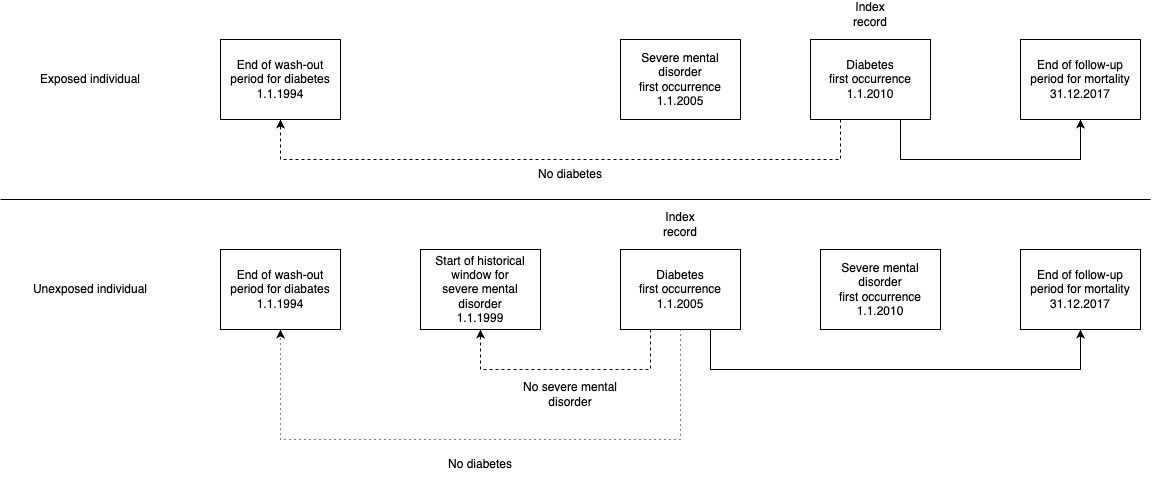


## Supplementary Figure 2 Survival probability with 95% confidence interval following the first hospitalization for atrial fibrillation in people with and without severe mental disorder

## Supplementary Figure 3 Survival probability with 95% confidence interval following the first hospitalization for cancers in people with and without severe mental disorder

## Supplementary Figure 4 Survival probability with 95% confidence interval following the first hospitalization for chronic kidney disease in people with and without severe mental disorder

## Supplementary Figure 5 Survival probability with 95% confidence interval following the first hospitalization for chronic liver disease in people with and without severe mental disorder

## Supplementary Figure 6 Survival probability with 95% confidence interval following the first hospitalization for chronic pulmonary diseases in people with and without severe mental disorder

## Supplementary Figure 7 Survival probability with 95% confidence interval following the first hospitalization for chronic viral hepatitis in people with and without severe mental disorder

## Supplementary Figure 8 Survival probability with 95% confidence interval following the first hospitalization for connective tissue disorders in people with and without severe mental disorder

## Supplementary Figure 9 Survival probability with 95% confidence interval following the first hospitalization for diabetes mellitus in people with and without severe mental disorder

## Supplementary Figure 10 Survival probability with 95% confidence interval following the first hospitalization for diseases of the circulatory system in people with and without severe mental disorder

## Supplementary Figure 11 Survival probability with 95% confidence interval following the first hospitalization for diseases of the endocrine system in people with and without severe mental disorder

## Supplementary Figure 12 Survival probability with 95% confidence interval following the first hospitalization for diseases of the gastrointestinal system in people with and without severe mental disorder

## Supplementary Figure 13 Survival probability with 95% confidence interval following the first hospitalization for diseases of the neurological system in people with and without severe mental disorder

## Supplementary Figure 14 Survival probability with 95% confidence interval following the first hospitalization for diseases of the urogenital system in people with and without severe mental disorder

## Supplementary Figure 15 Survival probability with 95% confidence interval following the first hospitalization for diverticular disease of the intestine in people with and without severe mental disorder

## Supplementary Figure 16 Survival probability with 95% confidence interval following the first hospitalization for epilepsy in people with and without severe mental disorder

## Supplementary Figure 17 Survival probability with 95% confidence interval following the first hospitalization for heart failure in people with and without severe mental disorder

## Supplementary Figure 18 Survival probability with 95% confidence interval following the first hospitalization for hypertension in people with and without severe mental disorder

## Supplementary Figure 19 Survival probability with 95% confidence interval following the first hospitalization for infectious and parasitic diseases in people with and without severe mental disorder

## Supplementary Figure 20 Survival probability with 95% confidence interval following the first hospitalization for inflammatory bowel disease in people with and without severe mental disorder

## Supplementary Figure 21 Survival probability with 95% confidence interval following the first hospitalization for ischemic heart disease in people with and without severe mental disorder

## Supplementary Figure 22 Survival probability with 95% confidence interval following the first hospitalization for multiple sclerosis in people with and without severe mental disorder

## Supplementary Figure 23 Survival probability with 95% confidence interval following the first hospitalization for Parkinson’ s disease in people with and without severe mental disorder

## Supplementary Figure 24 Survival probability with 95% confidence interval following the first hospitalization for peripheral artery occlusive disease in people with and without severe mental disorder

## Supplementary Figure 25 Survival probability with 95% confidence interval following the first hospitalization for prostate disorders in people with and without severe mental disorder

## Supplementary Figure 26 Survival probability with 95% confidence interval following the first hospitalization for stroke in people with and without severe mental disorder

## Supplementary Figure 27 Survival probability with 95% confidence interval following the first hospitalization for thyroid disorder in people with and without severe mental disorder

## Supplementary Figure 28 Survival probability with 95% confidence interval following the first hospitalization for tuberculosis in people with and without severe mental disorder

## Supplementary Figure 29 Survival probability with 95% confidence interval following the first hospitalization for ulcer or chronic gastritis in people with and without severe mental disorder

# References

1. Formánek T, Krupchanka D, Perry BI, Mladá K, Masopust J, Jones PB, et al. Risk of Mortality and Life-years Lost following Somatic Comorbidity in People with Pre-existing Severe mental disorderes: A National Cohort Study of Hospitalized Individuals in Czechia. 2023. <https://osf.io/5gfjv/>.

2. Formánek T, Krupchanka D, Mladá K, Winkler P, Jones PB. Mortality and life-years lost following subsequent physical comorbidity in people with pre-existing substance use disorders: a national registry-based retrospective cohort study of hospitalised individuals in Czechia. The Lancet Psychiatry. 2022; 9(12): 957-68.
